# The unity and diversity of verbal and visuospatial creativity: Dynamic changes in hemispheric lateralisation

**DOI:** 10.1002/hbm.26494

**Published:** 2023-09-29

**Authors:** Yixin Gao, Xinran Wu, Yuchi Yan, Min Li, Facai Qin, Mujie Ma, Xiaoning Yuan, Wenjing Yang, Jiang Qiu

**Affiliations:** ^1^ Key Laboratory of Cognition and Personality (SWU) Ministry of Education Chongqing China; ^2^ Faculty of Psychology Southwest University (SWU) Chongqing China; ^3^ Institute of Science and Technology for Brain‐Inspired Intelligence Fudan University Shanghai China

**Keywords:** creative thinking, dynamic laterality, prediction modelling, sparse canonical correlation analysis

## Abstract

The investigation of similarities and differences in the mechanisms of verbal and visuospatial creative thinking has long been a controversial topic. Prior studies found that visuospatial creativity was primarily supported by the right hemisphere, whereas verbal creativity relied on the interaction between both hemispheres. However, creative thinking also involves abundant dynamic features that may have been ignored in the previous static view. Recently, a new method has been developed that measures hemispheric laterality from a dynamic perspective, providing new insight into the exploration of creative thinking. In the present study, dynamic lateralisation index was calculated with resting‐state fMRI data. We combined the dynamic lateralisation index with sparse canonical correlation analysis to examine similarities and differences in the mechanisms of verbal and visuospatial creativity. Our results showed that the laterality reversal of the default mode network, fronto‐parietal network, cingulo‐opercular network and visual network contributed significantly to both verbal and visuospatial creativity and consequently could be considered the common neural mechanisms shared by these creative modes. In addition, we found that verbal creativity relied more on the language network, while visuospatial creativity relied more on the somatomotor network, which can be considered a difference in their mechanism. Collectively, these findings indicated that verbal and visuospatial creativity may have similar mechanisms to support the basic creative thinking process and different mechanisms to adapt to the specific task conditions. These findings may have significant implications for our understanding of the neural mechanisms of different types of creative thinking.

## Graphical abstract

This study applied sparse canonical correlation analysis to explore the dynamic lateralisation mechanisms of verbal and visuospatial creativity. We found that both common and different lateralisation mechanisms were involved in verbal and visuospatial creativity and that various brain networks were implicated in these processes.

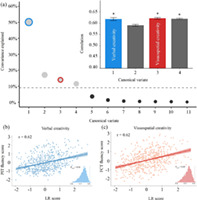

## 1 INTRODUCTION

Creativity can be defined as the capacity to generate output that is both original and appropriate (Runco & Jaeger, 2012; Sternberg & Lubart, 1996), which is considered as an essential factor for human survival and social development. By exploring the cognitive neural mechanisms of creativity, we can improve our understanding of and better cultivate creativity. Creative thinking is a complex cognitive function involving memory, language, problem solving and many other mental processes (Benedek et al., 2014; Madore et al., 2015; Zabelina et al., 2019), and the mechanisms behind it remain controversial and elusive because of the different measurement methods and tasks used in studies (Arden et al., 2010; Dietrich & Kanso, 2010). From the perspective of the product, creative thinking can be divided into verbal and visual forms (Lu et al., 2022). Verbal creative thinking (VCT) is the ability to generate novel and useful solutions through verbal forms, such as generating uncommon uses for a belt (Chrysikou et al., 2021), while visuospatial creative thinking (VSCT) is defined as generating the production of novel and useful visual forms, such as drawing (Aziz‐Zadeh et al., 2013). Although the measuring methods for both VCT and VSCT are similar, their underlying cognitive processes differ: verbal creativity tasks require participants to master sufficient knowledge, experience and necessary language processing abilities to selectively retrieve from memory and flexibly recombine information to generate innovative and appropriate ideas (Wu et al., 2005; Yang et al., 2022); in contrast, visuospatial creativity tasks depend more on individuals' ability to perform visual perception processing and spatial imagination, which is less influenced by knowledge background (Wu et al., 2005). Previous studies have revealed that VCT and VSCT have different neural mechanisms. VCT was found to rely more on the left hemisphere, including the prefrontal, superior temporal and inferior parietal cortex, while VSCT activated the right middle and inferior frontal gyrus, left precentral gyrus and bilateral thalamus (Boccia et al., 2015). Furthermore, VCT was associated with the grey matter volume of the bilateral lingual gyrus, praecuneus and inferior frontal gyrus (Iraji et al., 2021; Zhang et al., 2016; Zhu et al., 2013), whereas VSCT performance can be predicted by the cortical thickness of the right middle temporal, left inferior frontal cortex and grey matter volume of the right temporal lobe (Cousijn et al., 2014; Hahm et al., 2019).

The role of hemispheric lateralisation in creativity has always been a classic but controversial issue in creativity research. Previous studies suggested that VCT might be right‐dominant due to the right hemisphere's specialisation in loose semantic processing (Beeman & Bowden, 2000; Kounios & Beeman, 2014), while others found that both the left and right hemispheres are involved in VCT (Lindell, 2011; Takeuchi et al., 2010). Similarly, some studies suggested that VSCT was right‐dominant process (Gansler et al., 2011; Kenett et al., 2015; Kowatari et al., 2009; Moore et al., 2009; Razumnikova & Volf, 2015) as the role of right fronto‐parietal cortex in visual processing (Gansler et al., 2011), while others emphasised the importance of the left hemisphere (Huang et al., 2013; Moore et al., 2009). Recent machine‐learning study found VCT and VCST differ in terms of lateralisation (Chen et al., 2019), with VCT depended on inter‐hemispheric cooperation, while VSCT was dominated by the right hemisphere. Overall, the results are inconsistent, and the association between lateralisation and creativity remains largely unknown.

Previous studies have already made progress in exploring the neural mechanisms of VCT and VSCT. However, the methods used to study the lateralisation of these two types of creative thinking can still be improved. Recent studies suggested the nature of creative thinking as a dynamic process (Girn et al., 2020), but these dynamic characteristics cannot be captured by traditional static analysis methods. Some have begun to shift their focus from static functional connectivity (SFC) to dynamic function connectivity (DFC) in creative thinking to track time‐varying brain signals (Beaty et al., 2016; Beaty et al., 2018; Li et al., 2017; Patil et al., 2021; Sakoğlu et al., 2010; Sun et al., 2020). Emerging evidence suggested that functional lateralisation is modulated by the external environment and task demands (Bassett et al., 2006; Cohen et al., 2015; Costanzo et al., 2015), implying that hemispheric lateralisation is a time‐varying adaptive process. To quantify the time‐varying lateralisation features, a recent study applied DFC approaches and explored dynamic lateralisation from two dimensions, laterality fluctuations (LF) and laterality reversal (LR). The results showed that LF, but not LR, was positively correlated with language and cognitive flexibility (Wu et al., 2022). These dynamic laterality indices can help capture the subtle changes in laterality between two hemispheres that were omitted in previous studies using SFC approaches. Therefore, we expect to determine similarities and differences in the dynamic lateralisation mechanisms between VCT and VSCT.

In the present study, we hypothesised that VCT and VSCT exhibit both commonalities and distinctions in the mechanisms of dynamic lateralisation. To examine this hypothesis, resting‐state fMRI was used in this study. Additionally, four behavioural tasks were conducted outside the scanner to measure creative thinking. Specifically, we combined scores from four creative thinking tasks with 11 dimensions of the brain laterality dynamic. We utilised sparse canonical correlation analysis (SCCA) to identify hidden cognitive‐brain association patterns by combining creative thinking tasks and dynamic laterality index, to reduce overfitting problems in neuroimaging studies with high‐dimensional data (Smith & Nichols, 2018; Wang et al., 2020; Lee et al., 2020). Our goal was to explore similarities and differences in the dynamic lateralisation mechanisms between VCT and VSCT. Based on previous studies, two hypotheses were tested in this study. The first hypothesis pertained to the brain network distribution under VCT and VSCT. Some networks, such as the default mode network (DMN) and fronto‐parietal network (FPN), which were confirmed to be important for idea generation and evaluation (Beaty et al., 2021), are expected to equally contribute to both VCT and VSCT. Conversely, networks such as the language network (LN) and visual network (VN) may contribute more to one of the creativity types. The second hypothesis was related to hemispheric dominance, with a previous study employing AI suggesting that VSCT was right‐dominant while VCT relied on both left and right hemispheres. We assumed that using the dynamic lateralisation approach would find similar results.

## 2 METHODS

### 2.1 Participants

The present study utilised two undergraduate student datasets (main dataset: *N* = 597, validation dataset: *N* = 804). The main dataset was from an ongoing research project, the Gene‐Brain‐Behaviour project, a large sample dataset containing multiple behavioural variables and brain imaging data. Participants were recruited from Southwest University in a variety of ways, including the campus network, advertisements on leaflets and face‐to‐face communications on campus. Prior to study enrolment, each participant underwent a screening process encompassing self‐reported questionnaires alongside structured and semi‐structured interviews as part of exclusionary measures. All participants were required to be healthy and right‐handed with no history of psychiatric disorder, cognitive disability, substance abuse and MRI contraindications. Further details were described in the previous study (Chen et al., 2018). In addition, an external validation dataset from another ongoing research project, the Behavioral Brain Research Project of Chinese Personality (BBP), was used to test the generalisability of the results obtained from the main dataset. Same as the main dataset, all participants in the validation dataset were right‐handed, and none of them had a history of psychiatric or neurological illness.

In the main dataset, participants who both performed all behavioural assessments of creative thinking outside the scanner and underwent resting‐state scans were included. Additionally, participants with excessive head movement (mean frame‐wise displacement [FD] > 0.3) during resting‐state fMRI were excluded. A total of 597 right‐handed participants (mean age = 19.54 ± 1.46 years; range: 16–26 years; 173 males and 424 females) met the inclusion criteria. All participants provided written informed consent and received proper payment, and the research protocol was approved by the ethics committee of the review committee of the Brain Imaging Centre of Southwest University. In the validation sample, 804 participants were selected using the same criteria as those used for the main dataset (mean age 18.96 ± 0.99 years; range: 16–26 years; 260 males and 544 females).

### 2.2 Assessments of creative thinking

#### 2.2.1 Creative thinking tasks

We aimed to measure VCT and VSCT by using several creative thinking tasks. All these behavioural tasks were performed outside the scanner. To align with a previous study on hemispheric lateralisation mechanisms of VCT and VSCT that employed asymmetric index (Chen et al., 2019), we selected the same four creative thinking tasks: alternative use task (AUT), product improvement task (PIT), figural creativity test (FCT) and divergent thinking of figure (DTF). Prior to each task, participants were instructed to generate as many novel and unusual ideas as possible within the given timeframe, with instructions and tasks presented on a computer screen via E‐prime software; an answer sheet was provided for participants to record their ideas.

In the AUT, participants were presented with a commonplace object word and instructed to generate as many novel and unusual uses as possible within 3 min. Participants completed the AUT for two words (the word ‘brick’ and ‘can’) within a total duration of 6 min (Sun et al., 2016).

In the PIT, an image of elephant toy was presented, and participants were asked to think about how to improve this product, to make it more enjoyable, interesting and attractive within 10 min (Chen et al., 2018).

FCT was adapted from torrance tests of creative thinking (TTCT) for VSCT assessment in which 10 incomplete abstract figures were presented. Participants were asked to draw pictures using the 10 incomplete figures as a starting point within 10 min, and then give a title for each of them (Ye & Hong, 1988).

In the DTF, participants were asked to watch three intact figures which were composed of ambiguous line drawings. Participants were instructed to generate as many ideas as they could imagine that these figures could be within a total duration of 9 min (Creativity Testing Service, https://creativitytestingservices. com; 3 min per item).

In the main dataset, four trained raters assessed these tasks via three dimensions of creativity: (a) fluency, the number of ideas generated by each participant for a given item, excluding those that were difficult to comprehend, inappropriate or repetitive; (b) originality, which was defined as the novelty and unusualness of an idea and scored on a scale of 1 (not at all creative) to 5 (very creative) based on a scoring criterion table and raters' subjective assessment and (c) flexibility, the number of different categories of the ideas generated by a participant (for example, using bricks in the crafting of sculptures and collage art both belong to the same category of arts and crafts, whereas using bricks for swatting a fly would be classified under a different category; Long, 2014; Takeuchi et al., 2010). The fluency and originality scores were assessed for all four tasks, and flexibility scores were assessed in the AUT, PIT and DTF. We calculated the Cronbach's alpha coefficient among four raters to represent inter‐rater reliability for each creative thinking task (Cseh & Jeffries, 2019). The analysis of rater agreement showed good inter‐rater reliability, ranging from 0.91 to 0.96 in the main dataset. So we used the mean values of the four raters to represent individual creativity in each task dimension. Similarly, three raters assessed the tasks according to the same criteria in the validation dataset, and the analysis of rater agreement showed good inter‐rater reliability as well, ranging from 0.83 to 0.98 (inter‐rater reliability for each task in two datasets shown in Figure S1). Mean values among the three raters were calculated for each participant in each task dimension.

Overall, our study included 11 behavioural measures for creative thinking.

### 2.3 Image acquisition

In the main dataset, functional and structural data were obtained using a Siemens 3T Trio scanner (Siemens Medical System, Erlangen, Germany) at the Brain Imaging Centre of Southwest University. Resting‐state fMRI data (8 min) were obtained using a Gradient Eco type Echo Planar Imaging (GRE‐EPI) sequence: repetition time (TR) = 2000 ms, echo time (TE) = 30 ms, flip angle (FA) = 90°, field of view (FOV) = 220 × 220 mm^2^, slices = 32, thickness = 3 mm, interslice gap = 1 mm and voxel size = 3.4 × 3.4 × 4 mm^3^. High‐resolution, three‐dimensional T1‐weighted structural images were obtained using a magnetisation prepared rapid acquisition gradient‐echo (MPRAGE) sequence: TR = 1900 ms, TE = 2.52 ms, FA = 9°, slices = 176, FOV = 256 × 256 mm^2^, thickness = 1 mm and voxel size = 1 × 1 × 1 mm^3^.

In the validation dataset, the neuroimaging data were acquired on a 3T Prisma Siemens Trio scanner at Southwest University. Resting‐state fMRI scans (8 min) were obtained using a GRE‐EPI sequence: TR = 2000 ms, TE = 30 ms, FA = 90°, FOV = 224 × 224 mm^2^, slices = 62, thickness = 2 mm, interslice gap = 0.3 mm and voxel size = 2 × 2 × 2 mm^3^. Structural scans were obtained using a MPRAGE sequence: TR = 2530 ms, TE = 2.98 ms, FA = 7°, slices = 192, FOV = 224 × 256 mm^2^, thickness = 1 mm and voxel size = 0.5 × 0.5 × 1 mm^3^.

### 2.4 Image preprocessing

The resting‐state images were pre‐processed using the CONN toolbox (Whitfield‐Gabrieli & Nieto‐Castanon, 2012). The preprocessing of images included six steps. First, slice timing was performed to correct the time shift, and outlier scans were regressed out due to head motion during motion estimation. Specifically, potential outlier scans are identified from the observed global BOLD signal and the amount of subject‐motion in the scanner. Acquisitions with FD above 0.9 mm or global BOLD signal changes above 5 s.d. are flagged as potential outliers. Then, the images were segmented into grey matter, white matter (WM) and cerebrospinal fluid (CSF) tissue and normalised to standard MNI space (Ashburner & Friston, 2005). A Gaussian kernel of 8 mm FWHM was applied to smooth the images. The images were then denoised using the anatomical component‐based correction (aCompCor) method, and signals from WM, CSF and 12 head movement parameters were regressed out. After denoising, the images were linearly detrended, and a bandpass filter (0.01–0.1 Hz) was applied. Global signal (GS) regression was not applied to the present data because the mean hemispheric time series were used to calculate the lateralisation index (McAvoy et al., 2016).

### 2.5 Analysis methods

#### 2.5.1 Dynamic lateralisation index

We employed a recently developed method to measure the dynamic changes in lateralisation of hemispheres, which used a sliding time window based on a global signal to capture the time‐varying lateralisation architecture (Wu et al., 2022). Compared with the traditional method of measuring lateralisation using SFC (Liu et al., 2009), this new method took full advantage of the abundant dynamic properties in spontaneous brain activity, which were also essential for exploring creative idea generation (Agnoli et al., 2018; Wang et al., 2021). In this study, 12‐network parcellation with 360 bilateral parcels was applied to define the nodes (Glasser et al., 2016). This parcellation divides the cortex into 12 specific functional networks: visual network 1 (Vi1), visual network 2 (Vi2), ventral multimodal network (VMN), somatomotor network (SMN), posterior multimodal network (PMN), orbito‐affective network (OAN), LN, fronto‐parietal network, dorsal attention network (DAN), default mode network, cingulo‐opercular network (CON) and auditory network (AN).

We extracted the BOLD time series of each region of interest (ROI) for each participant. The dynamic lateralisation index (DLI) was defined as the difference between an ROI's BOLD correlation with the GS of the left hemisphere and its correlation with the right hemisphere at each time window. The window length was 30 TRs with a step of 1 TR. DLI at the *t*th window is defined by:

$${\mathrm{DLI}}_{t}=r\;\left({\mathrm{ROI}}_{i},{\mathrm{GS}}_{L}\right)–r\;\left({\mathrm{ROI}}_{i},{\mathrm{GS}}_{R}\right),$$

where ROI_*i*_ represents the BOLD time series of ROI_*i*_, and GS_L_ and GS_R_ indicate the GS of the left and right hemispheres, that is, the average time series of all voxels within the left and right hemispheres. We applied Fisher's z‐transformation to each correlation coefficient. Specifically, each time window had a length of 30 TRs, with the first time window containing the BOLD signals from the first to the thirtieth TR. The DLI value was calculated within this time window using the formula mentioned above. Subsequently, the time window was moved forward with the length of 1 TR, with the second time window covering the BOLD signals from the second to the thirty first TR. This process was iterated, moving forward the time window by 1 TR each time, thereby generating the DLI series. We further characterised the laterality time series from two aspects: the time‐varying magnitude and the sign of laterality, by LF and LR separately. LF was defined as the standard deviation of the laterality time series, and LR referred to the number of zero crossings of laterality in two consecutive time windows (Wu et al., 2022). We obtained 360 values of LF and LR for each participant.

#### 2.5.2 Sparse canonical correlation analysis

SCCA was utilised to explore the relationships between the 11 creative thinking measures and the laterality index (LF and LR separately). This method aimed to obtain latent components that reflect multivariate patterns across neural organisation and creative thinking. Canonical correlation analysis (CCA) can evaluate two sets of variables simultaneously, maximising the linear correlation between them; therefore, it can establish solid relationships between neuroimaging and behavioural data (Wang et al., 2018). SCCA is an extension of CCA that effectively reduces the overfitting problem by encouraging exactly zero contributions from some variables in each variable dataset. Moreover, it helps facilitate the explanation of results (Lee et al., 2020; Witten et al., 2009). We expected to use SCCA to identify two types of creative thinking abilities (VCT and VSCT) from several creativity measures and build relationships with neuroimaging data. Compared with traditional linear regression, SCCA can construct more robust associations between behavioural and neuroimaging data and account for more variance, especially in datasets with many individuals. Thus, we aimed to employ SCCA to explore the dynamic lateralisation mechanisms of different types of creative thinking. In this study, the SCCA method was implemented with a reliable and robust R package (penalised multivariate analysis, PMA) from CRAN (Witten et al., 2009).

#### 2.5.3 Model selection

We followed the data processing pipeline recommended by Wang et al. (2020), which has been utilised in several other similar studies (Wong et al., 2021; Xia et al., 2018). The data matrices D (creative thinking task scores), R (laterality reversal) and F (laterality fluctuation) were constructed after being transformed to z scores. Prior to performing SCCA, confounding variables were regressed out using a general linear model. Specifically, the effects of age and sex were regressed out from the brain and behavioural data. In addition, the head motion effect (mean FD) was further regressed out from the brain data.

The penalty coefficient in the PMA package ranges from 0.1 to 0.9, where 0.1 represents a smaller number of features and 0.9 represents a larger number of features. To determine the optimal coefficient for the model, we performed a grid search from 0.1 to 0.9 in increments of 0.1. We sought to identify the hyperparameters that can maximise the canonical correlation coefficient of the first pair of variates across 100 resampled samples, each of which contained two‐thirds of the dataset. The average value of the correlation was used to assess the model.

#### 2.5.4 Permutation test

To evaluate the statistical significance of each canonical mode, we created a null distribution for each mode through permutation tests. First, we randomly shuffled the rows of creative thinking measures to break the link between a participant's neuroimaging data and behavioural data. Then, SCCA with the same hyperparameters was performed to construct a null distribution of canonical correlation coefficients after 5000 permutation tests. The *p* values were calculated as the number of null correlations from the permutated dataset that exceeded the correlation coefficient estimated on the original dataset over a total number of null correlations. To account for multiple comparisons, we used Bonferroni correction across the selected modes.

#### 2.5.5 Bootstrap analysis

To estimate the stability of brain or behavioural features in each significant mode, 5000 bootstrapped datasets were created by randomly resampling with replacement. The creative thinking features with a 95% confidence interval or the laterality index whose 99% confidence interval did not cross zero were deemed to be stable throughout bootstrapped samples (Wong et al., 2021).

#### 2.5.6 External validation

The same hyperparameters were applied in the BBP sample. To test the reliability of the behaviour‐brain associations, we compared the latent components from the validation dataset with results obtained from the main dataset.

## 3 RESULTS

### 3.1 Behavioural results

The mean scores assigned by the raters were employed to represent individual creativity across different dimensions. To provide an initial overview of participants' task performance, a detailed presentation of their performances in both datasets is provided in Table 1.

**TABLE 1:** Dimension scores of four tasks in two datasets.

Main dataset (*N* = 597)	AUT			PIT		
	Mean	SD	Range	Mean	SD	Range
Fluency	4.78	2.20	0.5–12.5	10.38	4.08	1.5–27
Originality	11.25	5.59	1–29	23.94	9.96	4.25–76.5
Flexibility	3.97	1.62	0.5–10.5	6.31	2.10	1–16.75

	FCT			DTF		
	Mean	SD	Range	Mean	SD	Range
Fluency	8.73	1.86	2–10	8.30	2.87	2.33–17.44
Originality	9.64	3.05	0.75–18.5	21.07	7.85	5.58–45.89
Flexibility	‐	‐	‐	6.72	2.07	2.25–13.78

Validation dataset (*N* = 804)	AUT			PIT		
	Mean	SD	Range	Mean	SD	Range
Fluency	4.06	1.53	1–9.44	10.41	3.67	1–23.5
Originality	10.62	4.39	1.94–26.58	26.67	9.62	1–62
Flexibility	3.36	1.12	0.89–7.22	6.48	2.00	1–14

	FCT			DTF		
	Mean	SD	Range	SD	Mean	SD
Fluency	8.15	1.61	2–10	7.52	2.41	1–15.67
Originality	15.69	3.52	2–23.67	19.41	6.96	2.33–44.56
Flexibility	‐	‐	‐	6.52	1.86	1–12.44

Abbreviations: AUT, alternative use task; DTF, divergent thinking of figure; FCT, figural creativity test; PIT, product improvement task.

Before SCCA, each dimension score was transformed into *z* value (Figures S2 and S3).

### 3.2 Canonical modes between creative thinking tasks and dynamic lateralisation index

**FIGURE 1:**
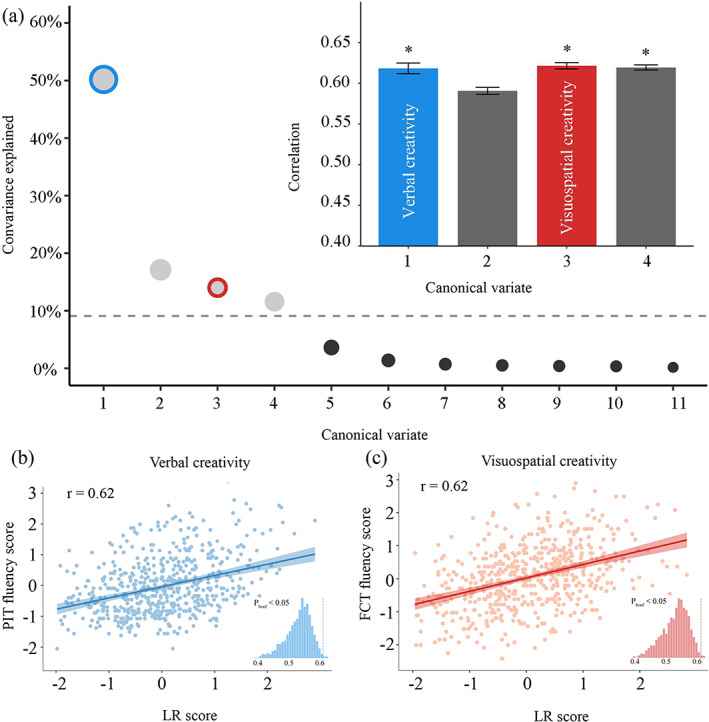
SCCA revealed multivariate patterns of linked dimensions of creativity measures and LR. (a) The first four canonical variates were selected based on the covariance explained. The dashed line marks the average covariance explained. Three modes survived the permutation, and two of them were meaningful. (b, c) Scatter plots represent the correlated patterns of LR and creative thinking measure with highest loading in each mode. Each insert displays the null distribution of SCCA correlation by permutation testing. The dashed line marks the actual correlation. LR, laterality reversal; SCCA, sparse canonical correlation analysis.

SCCA was employed as the model‐selection strategy. Based on the screen plot of covariance explained, a model of four modes with penalty levels of 0.9 on the LR and 0.9 on the creative thinking measures was obtained (Figure 1a). The first four modes accounted for 92.8% of the variance. Modes 1, 3 and 4 survived the permutation test (Mode 1: *r* = 0.619, *p*_bonf_ = .026; Mode 3: *r* = 0.621, *p*_bonf_ = .011; Mode 4: *r* = 0.618, *p*_bonf_ = .026). The same process was applied to LF, while very few creative thinking measures survived the bootstrap (loading coefficients for most creative thinking measures were zero). Thus, only LR was used for further analysis.

In SCCA, each mode can be conceptualised as a latent variable encompassing loadings associated with distinct creativity dimensions. Theses loadings within a specific mode represent the contribution of the creativity dimensions. Based on our hypothesis and previous research (Chen et al., 2019), it is expected that VCT encompasses AUT, PIT and DTF, whereas VSCT is anticipated to comprise FCT. As shown in Table 2, Modes 1 and 3 were found to exactly contain all 11 creative thinking measures with no repetition. Modes 2 and 4 displayed undistinguished patterns in their loading distributions and lack practical significance. Consequently, further analysis and discussion were performed only in Modes 1 and 3. Specifically, Mode 1 consisted of fluency, flexibility and originality of AUT, PIT and DTF, which were classical tasks measuring VCT in TTCT; therefore, Mode 1 was defined as ‘VCT’. Mode 3 included the fluency and originality of FCT, which were developed to measure VSCT in TTCT. Thus, Mode 3 was termed ‘VSCT’. These results were consistent with previous research that performed factor analysis on the same four creative thinking tasks (Chen et al., 2019). Each canonical variate represented a distinct pattern that associated a weighted set of creative thinking measures with a weighted set of LRs. For visualisation purposes, the correlation between the most heavily weighted creative thinking measure and LR was presented for VCT and VSCT. Specifically, the fluency score of PIT had the highest loading in Mode 1, while the fluency score of FCT was the most heavily weighted measure in Mode 3 (Figure 1b,c). The loadings of each creative‐thinking measure in VCT and VSCT were also presented (Table 2).

**TABLE 2:** Loadings of creative thinking measures in the main dataset.

	Mode 1	Mode 2	Mode 3	Mode 4
AUT fluency	0.30	0.36	0	−0.35
AUT originality	0.28	0.32	0	−0.31
AUT flexibility	0.34	0.33	0	−0.35
PIT fluency	0.40	−0.4	0	0
PIT originality	0.35	−0.4	0	0
PIT flexibility	0.37	−0.44	0	0
FCT fluency	0	0	0.69	0
FCT originality	0	0	0.68	0
DTF fluency	0.32	0	0	0.44
DTF originality	0.33	0	0	0.43
DTF flexibility	0.31	0	0	0.44

Abbreviations: AUT, alternative use task; DTF, divergent thinking of figure; FCT, figural creativity test; PIT, product improvement task.

**FIGURE 2:**
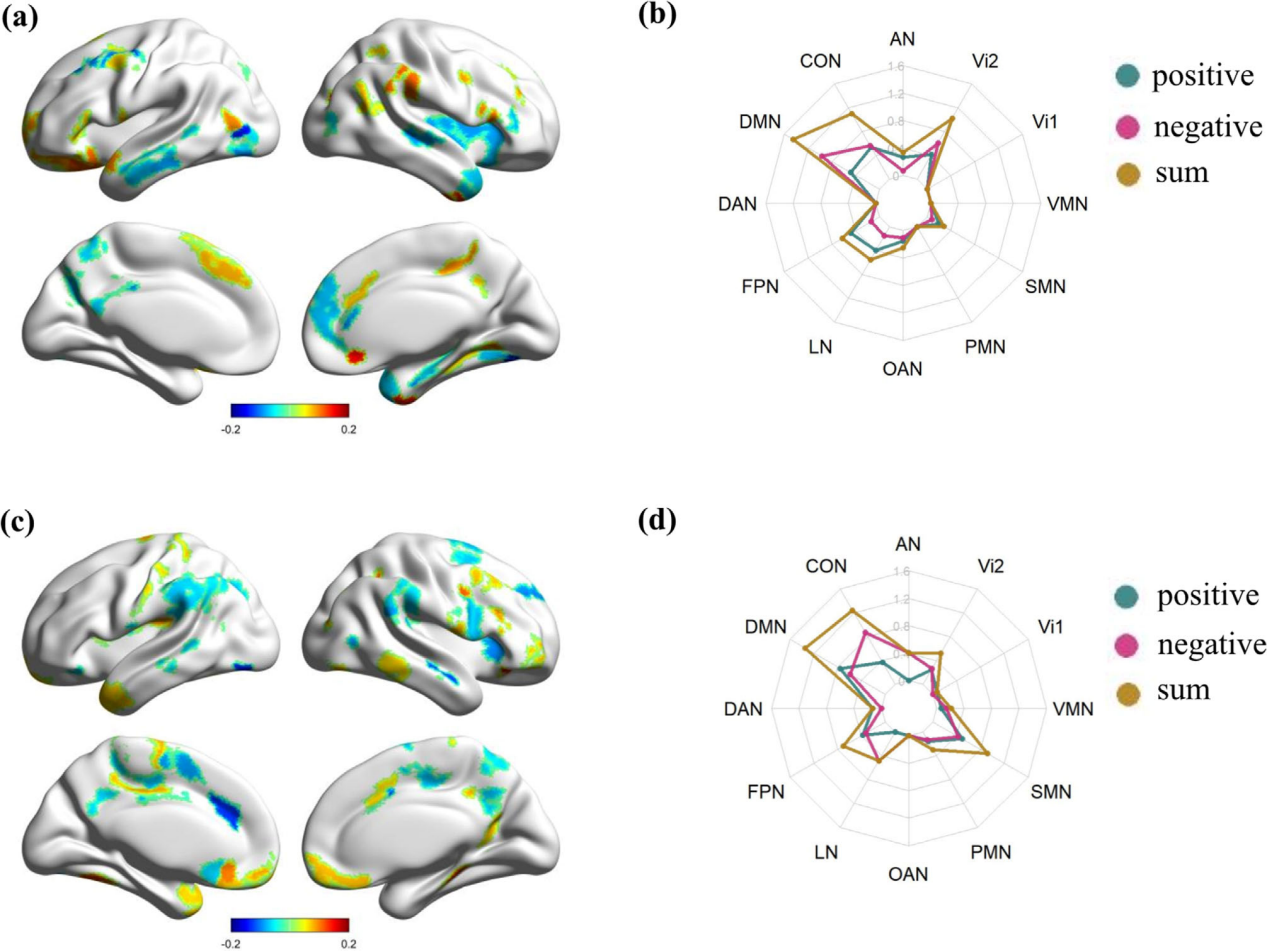
Correlation patterns between LR scores and creative thinking dimensions. (a, b) VCT: (a) represents brain regions with loadings in the main networks, (b) represents the absolute value of positive, negative and sum of loadings in 12 networks; (c, d) VSCT. AN, auditory network; CON, cingulo‐opercular network; DAN, dorsal attention network; DMN, default mode network; FPN, fronto‐parietal network; LN, language network; LR, laterality reversal; OAN, orbito‐affective network; PMN, posterior multimodal network; SMN, somatomotor network; ViN1, visual network 1; ViN2, visual network 2; VCT, Verbal creative thinking; VMN, ventral multimodal network; VSCT, visuospatial creative thinking.

In the VCT component (Mode 1), more loadings were negative among the brain regions that survived the bootstrap. The absolute value of loadings indicated the degree to which an ROI was associated with VCT. We calculated the sum of the absolute values of the loadings for positive and negative ROIs within each corresponding brain network. Our results showed that the DMN, CON, Vi2, FPN and LN had higher absolute values of loadings, indicating that these networks were more important for VCT. Furthermore, we compared the absolute value of loadings between positive regions and negative regions. We observed that LR in CON, DMN and Vi2 were negatively correlated with creative measures, while FPN and LN were dominated by positive loadings (Figure 2a,b). Regarding the hemispheric distribution, the loadings of the left hemisphere were similar to the right hemisphere for most networks, except for FPN, which was left dominated.

In the VSCT component (Mode 3), we also summed the absolute values of loadings for each brain network. Our analysis showed that the DMN, CON, SMN, FPN and Vi2 were strongly associated with VSCT. Specifically, CON and Vi2 were dominated by negative values, while FPN, DMN and SMN were dominated by positive values (Figure 2c,d). In terms of hemispheric distribution, similar to VCT, the loadings of the left hemisphere were also close to the right hemisphere for most networks except FPN, which was right dominated.

### 3.3 Canonical modes between creative thinking tasks and LR in the validation sample

**FIGURE 3:**
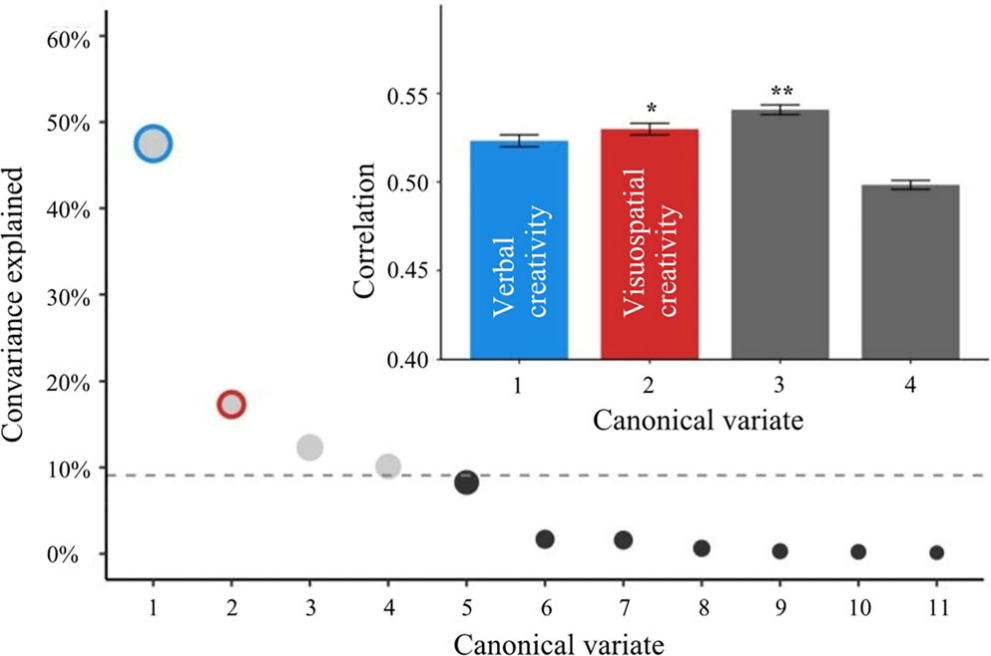
SCCA revealed multivariate patterns of linked dimensions of creativity measures and LR. The first four canonical variates were selected based on the covariance explained. The dashed line marks the average covariance explained. Only Mode 2 and Mode 3 survived the permutation, with Mode 1 showing an effect at uncorrected thresholds. LR, laterality reversal; SCCA, sparse canonical correlation analysis.

To evaluate the generalisability of our results, we performed the same analysis on the validation dataset. The screen plot of covariance explained revealed a model of four modes (Figure 3), with Modes 2 and 3 surviving the permutation test (Mode 1: *r* = 0.53, *p*_bonf_ = .048; Mode 2: *r* = 0.51, *p*_uncorrected_ = .037; Mode 3: *r* = 0.53, *p*_bonf_ = .03).

As shown in Table S1, Modes 3 and 4 displayed undistinguished patters in their loading distribution, lack practical significance and diverged from our hypothesis and previous research. In contrast, Modes 1 and 2 exhibited loading distributions that align with our hypothesis and possessed practical significance. As a result, we chose to focus our further analysis and discussion solely on Modes 1 and 2. Specifically, Mode 1 shared similar creative‐thinking measures with the VCT component of the main dataset, with higher loadings on fluency, flexibility of AUT, PIT and DTF, and originality of PIT and DTF. Therefore, we defined this mode as VCT in the validation dataset. Although Mode 2 did not survive the permutation test, it showed a significant effect at an uncorrected threshold, with higher loadings on fluency and originality of FCT, similar to the VSCT component in the main dataset. Thus, we labelled Mode 2 as VSCT in the validation sample. Distribution of each ROI was presented in Figure S4.

## 4 DISCUSSION

The present study revealed, for the first time, the similarities and differences between VCT and VSCT from the perspective of dynamic changes in hemispheric lateralisation. The results indicated that individuals with higher verbal creativity exhibited lower LRs in the CON, DMN and Vi2 and higher LRs in the FPN and LN. This finding suggested that less laterality switching between left and right in CON, DMN and Vi2 or more laterality switching in FPN and LN would boost verbal creativity. In contrast, individuals who had lower LRs in CON and Vi2 or higher LRs in DMN, FPN and SMN had better visuospatial creativity. This finding indicated that individuals who switched less in CON and Vi2 or switched more in DMN, FPN and SMN have higher visuospatial creativity. These results provide new insight into the unity and diversity of VCT and VSCT from the perspective of dynamic lateralisation.

Creative thinking is commonly divided into two categories: verbal creativity and visuospatial creativity. While previous research has explored the neural mechanisms underlying the difference between these two types of creativity using static hemispheric lateralisation (Chen et al., 2019; Wu et al., 2021), creative thinking is a dynamic process involving constantly changing spontaneous thought (Agnoli et al., 2018; Zedelius et al., 2021). Traditional static approaches averaged the BOLD time series and could not capture the time‐varying changes. Therefore, we employed the dynamic lateralisation method to explore the unity and diversity of verbal and visuospatial creativity. Specifically, we combined SCCA with machine‐learning methods to identify different modes of neuron‐cognitive patterns derived from the simultaneous decomposition of whole‐brain LR with creative thinking measures. Unlike traditional linear regression, SCCA incorporates multiple creative thinking tasks to obtain the scores of VCT and VSCT, resulting in more robust results and a stronger correlation between behavioural tasks and neuroimaging indices.

Our results suggested that the DMN, CON, Vi2 and FPN played a crucial role in the lateralisation component of both VCT and VSCT, indicating a shared neural mechanism for the two types of creative thinking ability. The DMN and FPN are well‐established brain networks involved in creative thinking. The executive theory of creativity (Benedek & Fink, 2019; Benedek et al., 2014) emphasises the contribution of strategic and controlled aspects of cognition required to manage and direct the creative thinking process (Beaty et al., 2021), which is supported by the DMN and FPN (Bendetowicz et al., 2018). Recent studies have found that cooperation between the FPN and DMN can predict individuals' verbal creativity (Beaty et al., 2021), as well as performance on visual artistic task (Ellamil et al., 2012). Similarity, studies employing static lateralisation methods also demonstrated the importance of the two brain networks for VCT and VSCT (Chen et al., 2019; Zhu et al., 2017). While CON and Vi2 have rarely been discussed in creativity studies, our results suggested their contribution to creative thinking. CON was associated with multiple task processing and executive control ability (Newbold et al., 2021; Wallis et al., 2015), and previous studies suggested that functional connectivity in CON could predict both individuals' figural creativity and verbal creativity (Sun et al., 2019; Wu et al., 2020). Vi2 is associated with individuals' figural creativity (Chen et al., 2019), with limited studies exploring the relationship between Vi2 and verbal creativity. However, a study applying brain network variability found that the variability between CON and the VN could predict verbal creativity (Sun et al., 2019), similar with our results. Additionally, some studies found that verbal creativity required internally‐focused attention, which was associated with the functional connectivity between right inferior parietal lobe and visual cortex, reflecting the inhibition on external input (Beaty et al., 2019; Benedek et al., 2016).

In addition, we also found the dynamic lateralisation mechanism of different creative thinking specificities: LN contributed to VCT, while SMN contributed to VSCT. With the anterior temporal lobe as the hub, the LN established connections with brain regions related to sensation and perception and forms semantic memory (Patterson et al., 2007). According to the spreading activation model in creativity, the generation of creative ideas started from an initial concept and forms long‐distance concepts through bottom‐up spontaneous thought and top‐down controlled retrieval (Collins & Loftus, 1975; Volle, 2018). Thus, LN is essential for VCT. SMN contributed to VSCT, which has been proven to be important for artistic aesthetics, motor execution, goal‐directed behaviour and planning underlying visuospatial creativity performance (de Manzano & Ullén, 2018; Pinho et al., 2015; Sacheli et al., 2022).

The results of present study also showed that the contributions of the left and right hemispheres were very close, which was partly consistent with previous studies confirming that verbal creativity required cooperation between the left and right hemispheres. However, a previous study found distinct results where VSCT was right‐dominant (Chen et al., 2019), which may result from the different brain indices we used. Chen focused on the balance between two hemispheres and investigated the dominant hemisphere in verbal and visuospatial creativity, while our study employed the LR to explore the switch of laterality, which represented the frequency of brain regions switched between left and right laterality, reflecting brain regions' communication with the contralateral hemisphere (Wu et al., 2022). Therefore, the similar distribution of the left and right hemispheres suggested that VCT and VSCT both required communication between the two hemispheres. However, it should be noted that our results suggested a differential role of the FPN in VCT and VSCT. Specifically, VCT was mainly supported by the left FPN, while VCST was supported by the right FPN. Early research highlighted the importance of the right hemisphere for figural creativity (Bhattacharya & Petsche, 2002; Miller et al., 2000; Miller & Hou, 2004), which may attenuate inhibitory processing from the left hemisphere (Mayseless et al., 2014). Consequently, the communication between the right FPN and the left hemisphere may also reflect this process. The left FPN, including the left inferior frontal gyrus and left superior parietal region, has been proven to be essential for VCT, indicating its crucial role in inhibiting proponent ideas during idea evaluation (Beaty et al., 2017). These findings suggested that most brain networks in VCT and VSCT had a similar hemispheric distribution, while FPN was left dominated in VCT and right dominated in VSCT. Therefore, we assumed that VCT may depend more on the left FPN's inhibitory processing of prepotent responses, while VSCT may depend more on the right FPN's attenuating inhibitory processing from the left hemisphere.

Our findings suggested that there may be differential contributions of shared brain networks to verbal and visuospatial creativity, as indicated by the observed patterns of positive and negative loading distributions. Specifically, we found that the LRs of CON and Vi2 were both negatively correlated with the behavioural component, while the FPN was positively correlated with creativity measures in VCT and VSCT. Moreover, the LR of the DMN was positively correlated with verbal creativity but negatively correlated with visuospatial creativity. The laterality index we applied (i.e., LR) represented extreme engagement with the contralateral hemisphere, which may hinder some common cognitive abilities (Wu et al., 2021). Therefore, the negative correlation in CON and Vi2 suggested that over‐engagement of contralateral hemisphere in these networks may interfere with their common function and thereby hinder creative thinking. In contrast, the positive correlation in the FPN indicated that it may be responsible for communicating more with the contralateral hemisphere to realise the top‐down control function. Additionally, the correlation between the DMN and creative thinking measures was positive in VCT and negative in VSCT. The positive correlation in the DMN may reflect complex semantic processing; thus, VCT can be speculated to have benefitted from the LR of the DMN. In contrast, VCST may need the DMN to cooperate less with the contralateral hemisphere to stably generate spontaneous thought.

Taken together, these results showed that VCT and VSCT shared some neural mechanisms while also displaying some differences in terms of dynamic lateralisation. Our use of the LR index, which combines hemispheric lateralisation and the DFC method, provides a new perspective for comparing the neural mechanisms of verbal and visuospatial creativity. Nevertheless, some limitations should be noted. First, the interpretation of the index is a challenge, and additional studies are necessary to validate our findings and explore the practical significance of the index. Second, our reliance on resting‐state fMRI data limited us to directly investigate the neural mechanisms during the creative thinking process. Third, we focused only on the temporal features of dynamic lateralisation, and further analysis could be conducted on the DLI, including spatial and temporal clustering. Future studies could design appropriate tasks and analyse task‐state fMRI data. Furthermore, exploring different lateralisation mechanisms between experts and novices could also make sense.

## 5 CONCLUSION

In summary, the present study combined dynamic lateralisation with SCCA to investigate the cognitive‐brain association patterns of VCT and VSCT. Our results indicated that both common and different lateralisation mechanisms were involved in VCT and VSCT and that various brain networks were implicated in these processes. Specifically, LRs of the DMN, FPN, CON and Vi2 were discovered in both VCT and VSCT, which could be considered the common lateralisation mechanism shared by the two types of creativity. In addition, VCT relied more on the LN, while VSCT relied more on the SMN, which could be a difference in the lateralisation mechanism between these types of thinking. In terms of hemispheric and loading distribution, these networks also presented unity and diversity under VCT and VSCT. These results provided novel insights into the longstanding controversial issue in creativity research and compensated for the ignorance of dynamic features in previous studies.

## FUNDING INFORMATION

This research was supported by the Major Research Plan of National Social Science Foundation of China (21&ZD312), National Natural Science Foundation of China (32071070, 32271112), Natural Science Foundation of Chongqing (cstc2020jcyj‐msxmX0299, cstc2021jcyj‐msxmX1138), the Research Program Funds of the Collaborative Innovation Center of Assessment towards Basic Education Quality at Beijing Normal University, 111 program (B21036), Fundamental Research Funds for the Central Universities (SWU2209239), Chang Jiang Scholars Program, National Outstanding Young People Plan and Chongqing Talent Program, Innovation Research 2035 Pilot Plan of Southwest University (SWUPilotPlan005).

## CONFLICT OF INTEREST STATEMENT

The authors declare to have no competing interests.

## Supporting information


**DATA S1.** Supporting Information.

## Data Availability

The data that support the findings of this study are available from the corresponding author upon reasonable request.
