# Supplementary material for: The unity and diversity of verbal and visuospatial creativity: Dynamic changes in hemispheric lateralisation
Source: Hum Brain Mapp. 2023 Sep 29;44(17):6031–42. doi: 10.1002/hbm.26494 (PMC10619400; doi:10.1002/hbm.26494)
Supplement: Supplementary file 1 — DATA S1. Supporting Information. [file HBM-44-6031-s001.docx]

**Supplementary Material**

**Supplementary tables**

| **TABLE S1** Loadings of creative thinking measures in the validation dataset | | | | |
| --- | --- | --- | --- | --- |
|  | **Mode 1** | **Mode 2** | **Mode 3** | **Mode 4** |
| AUT fluency | 0.31 | 0 | 0 | -0.52 |
| AUT originality | 0 | 0 | -0.31 | 0 |
| AUT flexibility | 0.31 | 0 | 0 | -0.5 |
| PIT fluency | 0.32 | 0 | 0.43 | 0 |
| PIT originality | 0.33 | 0 | 0.46 | 0 |
| PIT flexibility | 0.33 | 0 | 0.43 | 0 |
| FCT fluency | 0 | 0.65 | 0 | 0 |
| FCT originality | 0.09 | 0.66 | -0.27 | 0 |
| DTF fluency | 0.39 | -0.16 | -0.24 | 0.29 |
| DTF originality | 0.4 | -0.17 | -0.23 | 0.29 |
| DTF flexibility | 0.39 | -0.16 | -0.25 | 0.27 |

**Supplementary figures**

**
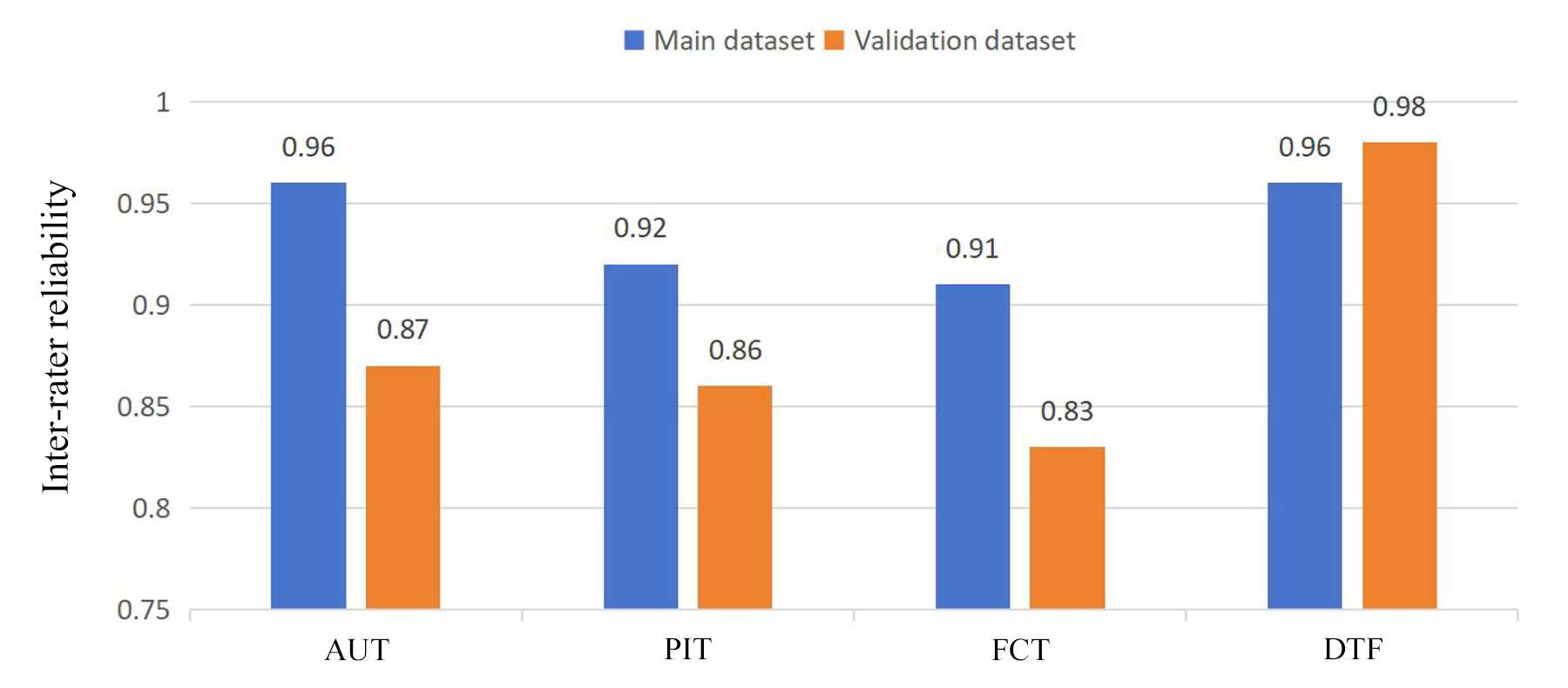
**

**FIGURE S1** Inter-rater reliability for each task in two samples. AUT, alternative use task; PIT, product improvement task; FCT, figural creativity test; DTF, divergent thinking of figure.

**
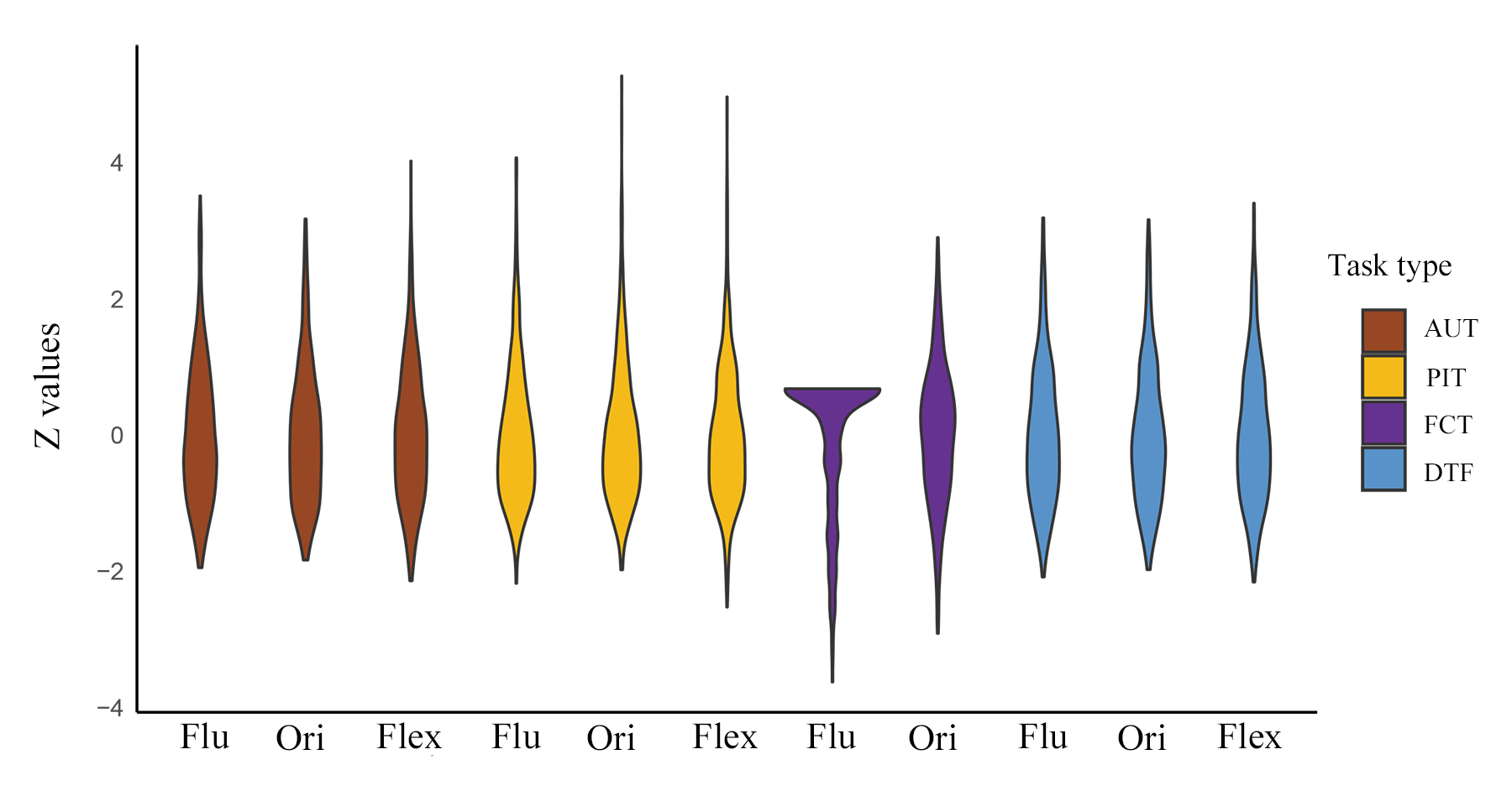
**

**FIGURE S2** Distribution of standardized scores for each dimension of four creative thinking tasks in the main dataset. Distinct colors were used to represent four different tasks. Flu, fluency; Ori, originality; Flex, flexibility.


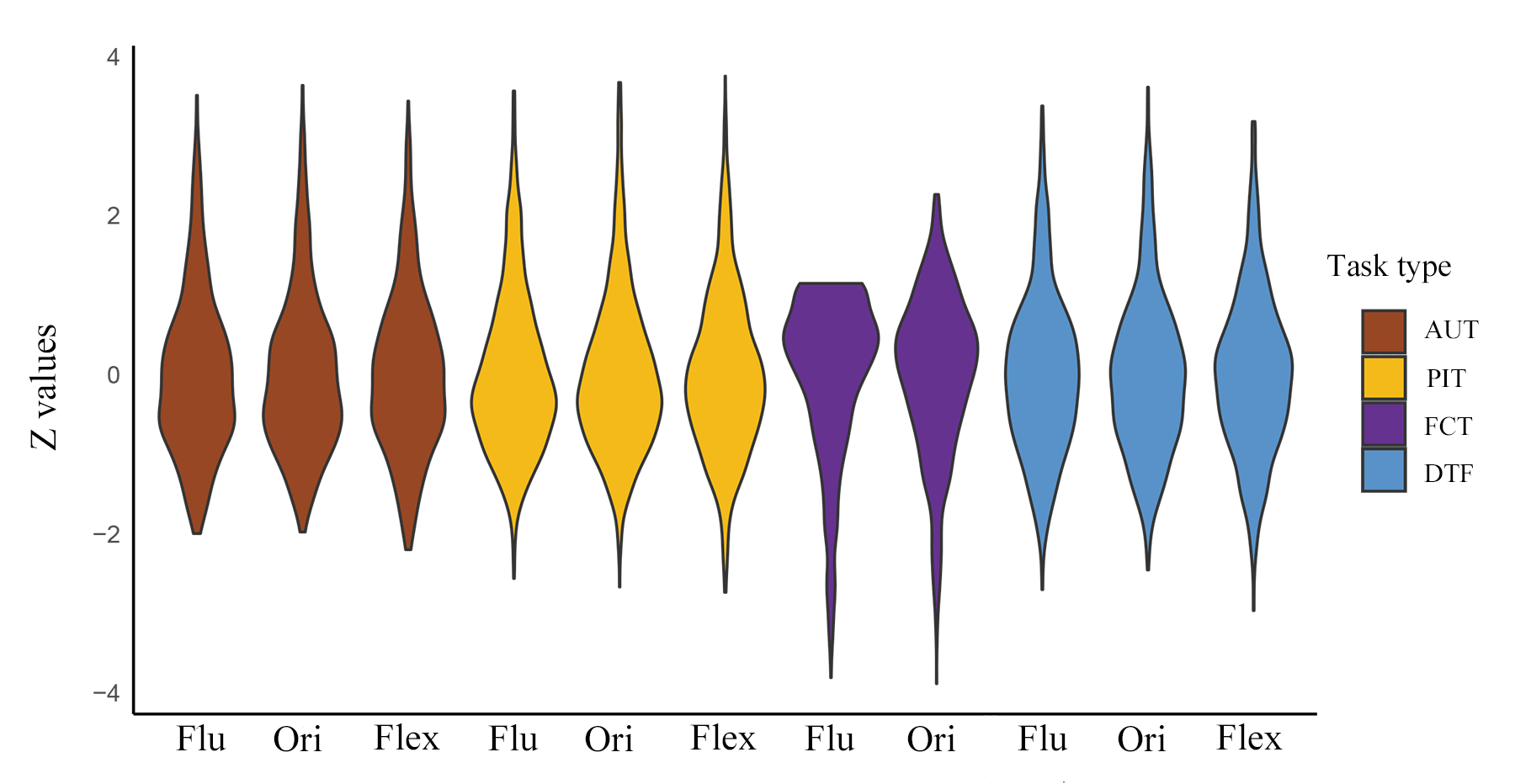


**FIGURE S3** Distribution of standardized scores for each dimension of four creative thinking tasks in the validation dataset. Distinct colors were used to represent four different tasks. Flu, fluency; Ori, originality; Flex, flexibility.

**
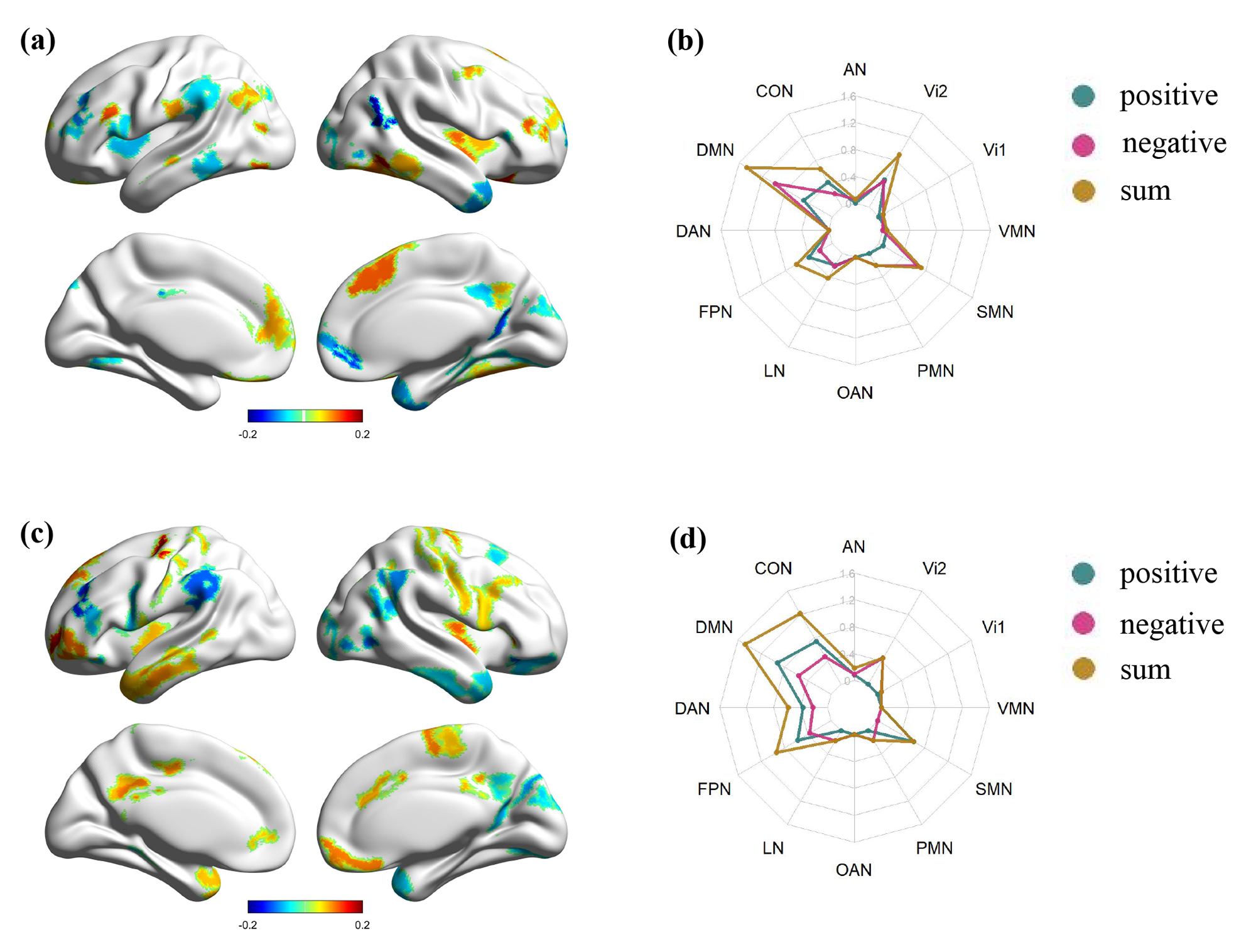
**

**FIGURE S4** Correlation patterns between LR scores and creative thinking dimensions. (a, b) VCT: (a) represents brain regions with loadings in the main networks, (b) represents the absolute value of positive, negative and sum of loadings in 12 networks; (c, d) VSCT.
